# Song duration mediates responses of territory owner in a songbird species with a small song repertoire

**DOI:** 10.1007/s10211-017-0257-4

**Published:** 2017-03-29

**Authors:** Tomasz S. Osiejuk, Aleksandra Jakubowska

**Affiliations:** Department of Behavioural Ecology, Institute of Environmental Biology, Faculty of Biology, Adam Mickiewicz University, Umultowska 89, 61-614 Poznań, Poland

**Keywords:** Song duration, Territory defence, Aggressive motivation, *Emberiza hortulana*, Playback experiment, Discontinuous song

## Abstract

Song is a sexually selected trait that is involved in mate attraction and territory defence in birds. Songs may convey information about different male quality components. They are flexible in terms of frequency, amplitude, and duration. Although changes in song duration are common, the function of this behaviour has been studied less strongly. It is known that song duration changes within a singing bout and may provide information about aggressive motivation. We tested whether the elongation and shortening of songs affects the responses of territorial ortolan bunting males to neighbour song playback. If changing song duration signals level of aggressiveness, then songs differing solely in duration may elicit behavioural responses of different strength. We performed two tests with different males assigned to two experimental groups and measured approaching and vocal response. In Experiment 1, we tested 18 males, which responded to the playbacks of elongated and normal (control) neighbour songs. In Experiment 2, we tested 17 males, which responded to the playbacks of shortened and normal (control) neighbour songs. Males responded significantly stronger to longer songs in both experiments as measured by the approach variable. Vocal response was not affected by treatment, but it was affected by the order of playback presentation. Our results indicate that song duration might be used for signalling current aggressive motivation during close interactions with rivals.

## Introduction

Song is a sexually selected trait that plays a key role in mate attraction and territory defence in birds (Catchpole and Slater 2008). It is a complex and flexible signal that often conveys information related to different male characteristics, such as quality, condition or current motivation (Gil and Gahr 2002). This flexibility is a result of multiple characteristics of song, which may be produced using different frequencies, amplitudes, durations, etc. The timing of singing appears to be very important, regardless of other dimensions of song variation. The majority of bird species are discontinuous singers and produce discrete song phrases separated by longer intervals of silence. Such song phrases usually belong to one or more categories, which form a so-called song-type repertoire and could be shared among males with diversified patterns (Catchpole and Slater 2008).

Song duration is among the most important dimensions of song variability, which may affect the responses of signal receivers. More singing per unit of time requires more energy for vocalization, and the overall duration and amplitude may signal the quality or condition of the signaller (Oberweger and Goller 2001). Birds may increase their singing effort by increasing their song rates (i.e., producing more phrases per time unit) or by elongating their song phrase duration (Cuthill and MacDonald 1990, Nelson and Poesel 2011). Song duration may vary for several reasons. For example, males of the great tit *Parus major* that are infected during development by the ectoparasite *Cerathophyllus gallinae* produce shorter songs later in life (Bischoff et al. 2009). In the barn swallow *Hirundo rustica*, males of different ages produce songs that are significantly different in terms of duration and structure (Galeotti et al. 2001). Experimentally stressed males of the whitethroat (*Sylvia communis*) produced shorter songs than males from a control group did (Borowiec et al. 2007). In the dark-eyed junco (*Junco hyemalis*), males use longer song types when they are more motivated to sing (Cardoso et al. 2009). Some studies also indicate sexual preferences of females for longer songs (e.g. Neubauer 1999, Gil et al. 2007). Such results indicate that song duration can carry information about male quality, current condition or motivation and could be important both for mate choice and territory defence.

Buntings from the *Emberiza* genus usually sing short songs and have small repertoires. In several species from this group, song strophes (i.e. discrete songs separated by usually longer pauses) consist of acoustically different parts (e.g. trills and whistles, Cramp and Perrins 1994). Males may modify song strophe duration by changing the strophe structures being used for each new performance, i.e. by omitting the whole trill phrase or whistles (e.g. in yellowhammer *Emberiza citrinella*; Rutkowska-Guz and Osiejuk 2004). However, often they are able to add or omit some syllables within a trill or trills, thus changing the strophe duration but keeping the structure and syntax of the song nearly unchanged. Such a characteristic of singing makes this group a good model for studying functionality of song duration in isolation from the structure of song. A surprisingly small number of studies have addressed questions related directly and solely to the function of song duration changes in such birds, while more research has focused on the meaning of different song structures within a song strophe (e.g. Gruber and Nagle 2010).

The ortolan bunting (*E. hortulana*) is a small Eurasian passerine species breeding in a wide spectrum of open habitats, including farmland. Males typically have a small repertoire of 2–3 song types, and within a local dialect area, males mostly share the final trill of a song (Cramp and Perrins 1994). Males announce their territories by singing from high song posts, and intrusion by a rival is usually met with a fast response from the territory owner. Approaching the intruder and switching from singing to uttering calls is a strong response to the invasion of a territory (Osiejuk et al. 2007a, 2007b). Such behaviour may end with a physical fight, especially during the first few days after arrival and especially when the behaviour is directed towards strangers (Skierczyński et al. 2007; Skierczyński and Osiejuk 2010). Males have been observed to change song duration, especially shortening of song, due to the counter singing of other males close to the territory or due to simulated territorial intrusion through the use of playback. However, this aspect of song variation has not been studied thoroughly in this species (Osiejuk et al. 2003; Łosak 2007).

The main aim of the present study was to test experimentally whether the duration of the song strophe (within the natural range of variation) affects the responses of territorial ortolan bunting males to a simulated playback intrusion. Earlier, we mostly observed song strophe duration changes during aggressive encounters and for shorter periods (usually few or dozen seconds) in comparison to regular singing from a song post which may last for many minutes (Łosak 2007, Skierczyński 2009). Therefore, we postulate that changes in song duration may provide information about the current aggressive motivation of males. Here, we asked if ortolan buntings also respond differently to songs of different durations. It is hard to predict a priori if shortened or elongated songs are more likely to be a signal of stronger threat. The existing studies show quite a large interspecific variation in response to songs of different duration with a predominance of species approaching playback faster when longer songs were presented (review in Nelson and Poesel 2012). These differences may be a result of the costs associated with song duration changes. It seems to be the most logical link between song duration and energy requirements. However, aggressive interactions are often very short and the cost of changing song duration could be negligible in comparison to other costs like proximity risk or retaliation (Bradbury and Vehrencamp 2011). Similarly, the energetic costs of acoustic signals increase with amplitude, but in some species the quiet signals are the best predictors of aggression and physical attack (e.g. Ręk and Osiejuk 2011). The experimental design applied in this study allowed us to test if birds are able to differentiate between the song strophes of different durations and how this changes their behaviour in the context of territory defence.

## Methods

### Study area and subjects

This study was conducted in farmland habitats that are located in Wielkopolska National Park in western Poland (coordinates of the centre of the study area: 52°17′N and 16°56′E). Ortolan buntings are common in this area and breed preferentially along forest edges and tree lanes surrounded by cultivated fields.

We recorded and analysed the song repertoires of 44 males in 2012 and 51 males in 2014 from the study area before we conducted any playback experiments. Each male was recorded for at least 10 min to capture his full repertoire. These males included the group of birds with which subsequent playback experiments were conducted and their neighbours, whose songs were later used as experimental stimuli.

### Playback equipment and song stimuli

For the playback experiments, we used an Apple iPod Classic 4th generation player (Shenzhen, China) with a wireless Sekaku WA-320 (Taichung, Taiwan) loudspeaker that had a 20-W amplifier (frequency range 50–15,000 Hz and a linear frequency response within the species-specific frequency range (i.e., 1.8–6.6 kHz)).

The songs used in the experiments were recorded in the field using a Marantz PMD661 (DandM Professional, Kanagawa, Japan) solid-state recorder coupled with a Sennheiser MKH70 microphone equipped with an MZW 60-1 basket wind shield and an MZH 60-1 long hairy cover (Sennheiser Electronic GmBH and Co. KG, Wademark, Germany) or a Telinga Pro-6 Twin Science parabolic microphone with a wind shield (Telinga, Botarbo, Tobo, Sweden). All of the songs used for playback were of good quality, recorded at short distances during windless mornings and free of noticeable background noise. The songs selected for playback were 2 kHz high-pass filtered (Avisoft SASLab Pro 5.x, Raimund Specht, Berlin, Germany) and then adjusted to match the amplitude level and envelope of natural songs (i.e. 86 dB SPL A-weighting at 1 m from the loudspeaker). The SPL value was set according to the amplitude level of typical ortolan bunting songs, which had previously been measured in the field using a CHY 650 (Ningbo, P.R. China) sound level meter. The amplitude manipulations were small and did not affect the song structure. All of the sounds recorded and used for playback were PCM WAVE files, with a 48 kHz sampling rate and a 16-bit resolution.

**Fig. 1:**
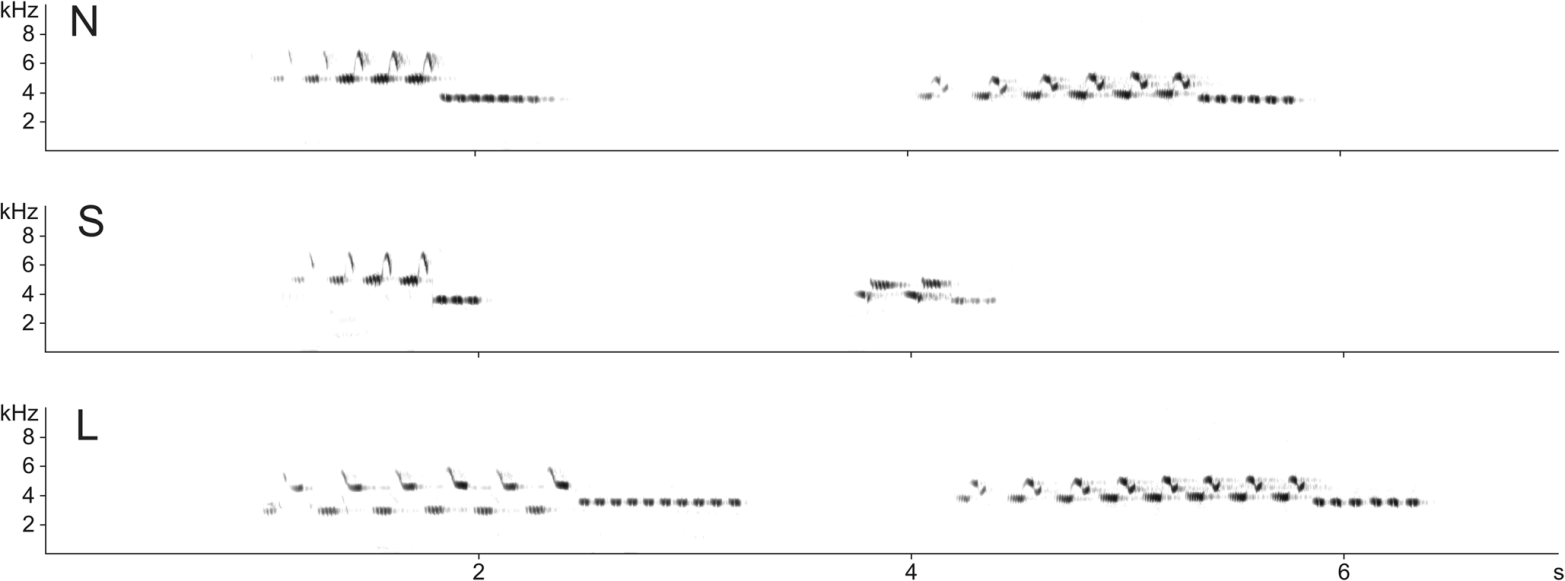
Spectrograms of exemplary songs of the ortolan bunting used for the playback experiments. Illustrated are two different song types used in treatments with normal song duration (N), shortened song duration (S), and elongated song duration (L). All song types shared the same local dialect final phrase

Initially, we prepared 140 different song renditions from 70 different males for the two playback experiments designed to test differences in responses between normal and elongated songs (Experiment 1) or normal and shortened songs (Experiment 2). Each song rendition belonged to one of two types of stimuli: (1) songs from an adjacent neighbour of a focal male (N-treatment or control) or (2) songs from the same adjacent neighbour that were slightly elongated (L-treatment) or shortened (S-treatment). Because most of the males had only two different song types in their repertoires (some had three), for the playback, we always selected exactly two of the most frequently sung song types from each male’s repertoire. In the case of N-treatment, the songs that were used were only manipulated to remove low-frequency noise and adjust the amplitude to the same level, as described above. As songs of the ortolan bunting consisted of two (initial and final) trilled phrases with repeated syllables (see Fig. 1), we used the following procedure for manipulating their durations. In the case of L-treatment, 2–5 syllables in the middle of the song were duplicated to increase the song duration. We always added syllables from both the initial and final song phrases. The manipulations were done in the middle of the song because the initial and final syllables have usually lower amplitude than those in the middle of the song. The manipulated songs were later amplitude adjusted and were longer than natural but still had a standardized maximal amplitude (86 dB SPL) and characteristic envelope within phrases. Analogously, in the case of S-treatment, 2–5 syllables from the middle of the song were removed before final amplitude adjustment. Because the syllables within the initial song phrase are longer than those from the final song phrase, we usually removed less initial syllables than final syllables and attempted to keep the proportions of both song parts the same as before the manipulations. The average duration of songs (±SD) used for particular treatments in both experiments differed significantly. In the case of Experiment 1, songs in L-treatment (2.2 ± 0.21 s) were significantly longer (*t*_34_ = −10.58, *p* < 0.001) than the songs in N-treatment (1.6 ± 0.20 s). In the case of Experiment 2, songs in S-treatment (1.0 ± 0.23 s) were significantly shorter (*t*_32_ = 10.60, *p* < 0.001) than the songs in N-treatment (1.66 ± 0.28 s). Simultaneously, the song duration of the playback samples used in N-treatments for the two experiments did not differ significantly (*t*_33_ = −1.663, *p* = 0.106). However, the durations of the manipulated songs were still well within the natural range of variation. As presented by Łosak (2007), in this population, males sing songs that vary in duration (±2 SD) from 1.04 to 1.91 s (these values come from measurements of over 12,000 songs). The song manipulation procedures are illustrated in Fig. 1. All of the manipulations were performed using the current version of Avisoft SASLab Pro 5.x.

### Playback experiment protocol

The experiments were conducted between 4 and 14 May 2012 and 2014, between 05:00 and 10:00 local time. Each male was subjected to one experiment (1 or 2) with two treatments. In experiment 1, males (*n* = 18) responded to the playback of elongated neighbour songs (L-treatment) and to the playback of normal neighbour songs (N-treatment) as a control. In experiment 2 (*n* = 17), males responded to shortened neighbour songs (S-treatment) and to normal neighbour songs (N-treatment) as a control. Each male responded twice to playback of the same neighbour, manipulated and natural. Each male was also tested with stimuli derived from repertoires of different males within the studied population. Ortolan buntings are known to respond stronger to the playback of a stranger song than to the song of a neighbour (Skierczyński et al. 2007, Skierczyński and Osiejuk 2010). Therefore, we used as playback stimuli songs of neighbours instead of strangers from the local population. We expected that simulated intrusion of neighbours singing phrases of different duration would be a better way to assess motivation encoded in a signal than by using stranger songs. By using stranger’s songs, the territory owner could respond very strongly regardless of song duration as a strange male is always perceived as a greater threat than a known neighbour is (Trivers 1971, Godard 1991). To minimize observer bias, blinded methods was used when all behavioural data were collected. The order of the treatments in both experiments was random and the song types used for each playback (treatment or normal) were drawn from a pool of encoded files. Therefore, we never knew before the first trial with a particular male which playback he would receive. The experimental period for each male was chosen based on behavioural observations to correspond to the time when unpaired males defended their territories intensively and after territorial borders with neighbours had been well established for at least a few days. All subject males and their neighbours had been recorded and their territories mapped (especially song post locations) prior to the experiments. Some were colour-ringed, but the confirmed identity of males was based on the analysis of the song repertoire and individuality of the specific frequency of initial song phrases (see Osiejuk et al. 2005, Osiejuk 2014). Each territory was visited at least every second day to record a target male and his neighbours and to evaluate the current social situation (e.g. pairing status, presence of neighbours, and spatial segregation of birds). The experiments were conducted only when the arrangement of the territories of male subjects and neighbours remained unchanged between the initial field inspection and the subsequent experiments (2–4 days later). This approach ensured that the songs used in experiments were derived from the repertoire of a well-known neighbour. The loudspeaker was fixed in a tree approximately 2 m above the ground before each experiment, and its location was changed slightly (5–10 m) between subsequent treatments with the same male to avoid habituation. In both treatments, songs were played back within the focal male territory from a place near the border of the neighbour male whose song was used in the N-treatment. The loudspeaker was always within the subject male’s territory and placed at a distance of 20–40 m from the song post used by the focal male during equipment set-up. We tried to place the loudspeaker in a way that allowed the focal male to fly towards it and to land in a tree or another elevated place at a distance of less than 1 m from the speaker. Each male could therefore approach the loudspeaker without getting closer to the observer, who was located perpendicular to and 20–30 m away from the loudspeaker–focal male line. The two treatments for each subject were conducted in a random order and were separated by a 1-day period. Experiments were only conducted if the subject males were singing regularly and the neighbour whose songs were used for stimuli preparation were silent and not present at typical song posts.

Each treatment consisted of three phases: a 2-min recording of a male just before playback started (PRE), a 2-min playback (PLAY) and a 2-min post-playback recording (POST). The behaviour of the focal male was observed, and his songs and calls were recorded during all phases. The vocalizations of the focal male were recorded using a Marantz PMD661 solid-state recorder coupled with a Telinga Pro-6 Twin Science parabolic microphone that was directed towards that male, and observations of his behaviour were dictated to an Olympus Ls-11 recorder by a second observer. Recordings and notes on behaviour were transcribed within a few days using the Raven Pro 1.4 software (Cornell Lab of Ornithology, Ithaca, USA), which enabled the extraction of response variables with ≤1 s accuracy.

Playback pattern (song rate and eventual variety) imitated natural singing (Osiejuk et al. 2003). Two song types were played back with a species-typical rate of six songs per minute during each PLAY phase of experiments. The first song type that was used was played six times during the first minute of PLAY, and the second song type was played back six times during the second minute of PLAY. The order of the use of particular song types for each male was random.

The following measures of the original response to playback were recorded: flight latency towards the loudspeaker (s); time spent within 5 m of the loudspeaker (s); closest approach to the loudspeaker (m); average song duration during and after playback (s) and the numbers of flights, songs and calls during and after playback. We used a Bushnell Yardage Pro Sport 450 laser rangefinder (1 m accuracy) for measuring distance between loudspeaker and the initial position of a tested male, and both tape measure and rangefinder for determining landmarks within the potential arena where a male might fly. We especially focused on crossing 5 m to loudspeaker and the closest distance to it, which sometimes required additional measurements to be taken after the experiment.

### Statistical analyses

Birds’ responses to territorial intrusion typically have a multidimensional character; therefore, we combined all original variables into orthogonal principal components using a principal component analysis (PCA). We obtained two principal components with eigenvalues over 1.0 that were correlated to the original variables and described the birds’ responses separating their vocalizations and movements (Table 1). We assessed the factorability of the data; according to the Bartlett test of sphericity (198.41, *P* < 0.001), the dataset should be considered appropriate. The Kaiser–Meyer–Olkin measure of sampling adequacy had a value of 0.680, and then the degree of common variance among the original variables was adequate (Tabachnick and Fidell 2013).

**Table 1:** Eigenvalues, variance explained and weightings of the original variables in the first two principal components extracted from the ten original variables of the response to the playback

Statistics and original response variable	Component	
	PC1—Approaching	PC2—Vocal response
Eigenvalue	2.784	1.870
% of variance	39.78	26.71
Cumulative %	39.78	66.49
Flight latency	**−0.86**	0.04
Time within 5 m distance	**0.54**	0.05
Closest distance	**−0.90**	0.06
Flights after playback start	**0.78**	−0.24
Songs after playback start	−0.04	**0.88**
Calls after playback start	0.57	**−0.62**
Average song duration after playback start	0.03	**0.80**

Measures that contributed most to the particular compound variable are in bold

We found no statistically significant differences when comparing a particular male’s pre-experiment distance to the loudspeaker between the L- and N-treatments in experiment 1 (paired *t* test, *t*_17_ = 1.83, *p* = 0.084) and between the S- and N-treatments in experiment 2 (paired *t* test, *t*_16_ = 0.91, *p* = 0.374). Similarly, we found no significant differences in the number of songs sung before playback began between the L- and N-treatments in experiment 1 (paired t test, *t*_17_ = −0.05, *p* = 0.960) and between the S- and N-treatments in experiment 2 (paired t test, *t*_16_ = −1.82, *p* = 0.088). Since the initial distance of a male to the loudspeaker depends on the spatial features of each territory (e.g., trees available and song post distribution) and cannot be standardized perfectly under field conditions, we included this variable in the analysis. We also used the number of songs sung by focal males and their average duration during PRE playback as another covariate, which should enable better model estimation if there is a relationship between song effort before simulated intrusion and the strength of a male’s subsequent response.

For both principal components of each experiment, we performed all possible (the null model, the full model and all possible combination of parameters) general linear mixed models (GLMM) with a Gaussian error distribution and an identity link function to test differences in response to playback between treatment and control. Male identity was included as a random intercept and the following variables were included as fixed parameters: treatment, order (treatment or control first) or covariates: distance to loudspeaker before playback begun, number of songs sung before playback, average song duration before playback. In the case of experiment 1, we removed a single outlier male from the analysis who was responding extremely weakly in both treatment and control.

To choose the best models, we used Akaike’s information criterion (*AIC*) calculated as *AIC* = −2ln(*L*) + 2 *k*, where *k* is the number of fitted parameters in the model including the intercept and *L* is the maximum likelihood estimated for the model. The ratios between the number of observations and the number of fitted parameters in the most complex models was below 40; therefore, we used a modified version AIC_c_ for small sample sizes (Burnham and Anderson 2002). To compare the best-fitted model with the lowest *AIC*_*C*_ (*AIC*_*Cbest*_) to any other model (*AIC*_*Ci*_), we calculated *∆AIC*_*Ci*_, where *∆AIC*_*Ci*_ = *AIC*_*Ci*_ *− AIC*_*Cbest*_. We present in this study only a set of models which had *∆AIC*_*C*_ < 2 value (Arnold, 2010; Burnham et al., 2011). For these models, we calculated Akaike’s weights (*w*_*i*_), which vary between 0 and 1 and could be interpreted as the probability that a given model is the best at approximating the data. We also present the evidence ratio (ER),which provides a measure of how much more likely the best model is than model *i* (*∆*_*best*_ is the *∆* value for the best model = 0). For example, if for second best model *ER* = 2, it means that the first model is approximately two times more likely to be the best approximating model than the second (Symonds and Moussalli 2011). All statistical analyses were calculated in STATA/SE 14.2 (StataCorp, College Station, TX, USA).

## Results

All of the approach-related response measures had stronger loadings on PC1. Higher PC1 values corresponded to stronger responses (i.e. males approached the speaker faster, closer, made more flights and stayed in close proximity to the speaker for longer periods) (Table 1). The vocal response measures had stronger loadings on PC2. Higher PC2 values corresponded to the number of songs sung and average song duration after playback began and was negatively related to the number of calls given (Table 1). PC2 is a good reflection of the typical vocal response of ortolan bunting males that are faced with territorial intrusion: they decrease song rate and start calling (e.g. Osiejuk et al. 2007; Osiejuk 2014; Table 1). Therefore, lower values of PC2 reflect stronger vocal response. Both compound measures of response were included as dependent variables into GLMM and all models with highest probabilities (Δ AIC_C_ < 2) are presented in Table 2.

**Table 2:** Models with highest probability (Δ AIC_C_ < 2) assessing variation in the ortolan bunting males’ response to playback

Model	AIC_C_	Δ AIC_C_	*w*_*i*_	ER
Experiment 1				
PC1 approaching				
T	97.25	0.00	0.33	
T + O	99.06	1.80	0.13	2.46
PC2 vocal approach				
T + O + D + SP + SDP	94.16	0.00	0.52	
T + O + D	95.56	1.40	0.26	2.01
T + O	96.03	1.87	0.20	2.55
T + O + D + SP	96.03	1.87	0.20	2.55
Experiment 2				
PC1 approaching				
T	90.06	0.00	0.69	
PC2 vocal approach				
T + O	101.06	0.00	0.48	
T + O + D	102.59	1.53	0.22	2.15

The Akaike weight (*w*_*i*_) and evidence ratio (ER) were calculated on the basis of Akaike’s information criterion corrected for small sample size (see the “Methods” section for details)

*T* treatment, *O* treatment/control order, *D* distance between male and loudspeaker before playback, *SP* number of songs sung during 2 min of recording before the playback, *SDP* average song duration before the playback

We found that in experiment 1, the treatment has the strongest effect on male approaching behaviour (PC1, Tables 2 and 3) and birds had a stronger response to elongated than to the normal duration songs (*β* ± SE = 0.70 ± 0.30, *p* = 0.022). Considering vocal response in experiment 1, we found no significant effect of the treatment on the PC2 in any of the presented models (Tables 2 and 3; all *p* > 0.18). This indicates that the duration of playback song in experiment 1 did not affect males’ vocal behaviour. However, all the best models showed significantly stronger vocal response (switching from singing to calling) when order of presentation of playback was elongated-normal song (Table 3, *β* ± SE = 0.84 ± 0.27, *p* = 0.002 for the best model).

**Table 3:** Estimates with standard errors and significance for predictor variables used in models with highest probability (Δ AIC_C_ < 2) assessing variation in the ortolan bunting males’ response to playback

Model	β (SE) and *P* values for predictor variables				
Experiment 1	T	O	D	SP	SDP
PC1 approaching					
T	0.70 (0.30) *p* = **0.022**				
T + O	0.70 (0.30) *p* = **0.022**	−0.24 (0.30) *p* = 0.433			
PC2 vocal approach					
T + O + D + SP + SDP	0.06 (0.26) *p* = 0.815	0.87 (0.27) *p* = **0.002**	−0.02 (0.01) *p* = **0.02**	-0.01 (0.02) *p* = 0.633	0.86 (0.37) *p* = **0.019**
T + O + D	0.24 (0.29) *p* = 0.408	0.93 (0.28) *p* = **0.001**	−0.02 (0.01) *p* = 0.074		
T + O	0.38 (0.29) *p* = 0.186	1.00 (0.29) *p* = **0.001**			
T + O + D + SP	0.22 (0.28) *p* = 0.425	0.92 (0.27) **0.001**	−0.02 (0.01) *p* = **0.046**	0.02 (0.02) *p* = 0.211	
Experiment 2					
PC1 approaching					
T	−0.84 (0.27) **0.002**				
PC2 vocal approach					
T + O	0.16 (30) *p* = 0.584	−0.74 (0.33) **0.023**			
T + O + D	0.17 (30) *p* = 0.565	−0.73 (0.33) *p* = **0.024**	0.003 (0.02) *p* = 0.822		

*T* treatment, *O* treatment/control order, *D* distance between male and loudspeaker before playback, *SP* number of songs sung during 2 min of recording before the playback, *SDP* average song duration before the playback

Significant effects (*p* < 0.05) of predictors are in bold

In experiment 2, in which males responded to normal and shortened songs, we found that treatment had the strongest effect on approaching (PC1) and all the models which contained more factors were substantially worse (Δ AIC_C_ > 2) and are not presented in Table 2. We found that males approached the loudspeaker more slowly and stayed farther from it during S-treatment than during the control (Table 3, *β* ± SE = −0.84 ± 0.27, *p* = 0.002). In experiment 2, we also found no significant differences in overall vocal response (PC2) between treatment and control (Table 3). The best-fitted model included treatment, order and distance variables (Table 2), but only the order effect was significant (Table 3). Males were found to respond significantly stronger (more calls and decrease of singing) when the order of playback presentation was a normal-shortened song (Table 3, *β* ± SE = −0.74 ± 0.33, *P* = 0.023 for the best model).

## Discussion

Our results support the hypothesis suggesting that changes in song duration could reflect the motivation of the rivals involved in a territorial conflict. We found that ortolan buntings responded more strongly to long than normal songs (experiment 1) and less strongly to short than normal song (experiment 2) as measured by approaching response (PC1). Therefore, males responded faster and moved closer to the loudspeaker when longer songs were presented. This result well reflects our hypothetical expectation that song duration may mediate a response of the rival male and that songs with different durations should evoke opposite responses. As for the vocal response variable (PC2), we found no significant effect of treatment in both experiments. However, we found in both experiments that males had a significantly stronger vocal response if the order of playback presentation started with longer songs.

Due to the observation of weaker approaching responses to shorter songs, it seems that singing with lower effort (shorter songs should cost less than long songs) signals a lower threat value. However, it is good to keep in mind that natural close-range interactions between males in this species last for a short time (usually less than 1–2 min, own observations) and the energy costs of singing are not likely to be very important here. It seems more probable that important costs are resulting from how the receiver of the signal evaluates the threat value of the invading rival and the probability of his aggressive response, including physical attacks (Bradbury and Vehrencamp 2011). In this light, one could argue that results of this study indicate that males refrain from approaching more threatening—i.e. shorter—signals (Searcy et al. 2000; Searcy and Beecher 2009). However, such an explanation also seems to be unlikely. In our experiments, we simulated intrusion of a known neighbour, that is, an individual which per definition is not a highest threat level (Fisher 1954). A stronger response to strangers than neighbours was earlier experimentally confirmed for the species with territory owners less likely to approach the speaker playing a neighbour’s song (Skierczyński et al. 2007; Skierczyński and Osiejuk 2010). Hence, the general pattern of response in neighbour–stranger discrimination experiments is very similar to the results presented here and the pattern of response to longer and shorter songs in both experiments. Therefore, we conclude that longer songs of a known neighbour are perceived as a higher threat and evoke a stronger response than do shorter neighbour songs. This study enables us to state that song duration alone modifies the response of territorial males. In some other studies, the manipulation of song duration consisted of eliminating one of the song strophe structures completely and likely affected functionally important units (e.g. final trill coding dialect identity; Nelson and Soha 2004). Thus, song duration changes per se might be an additional acoustic tool for maintaining a territory without the use of physical fights. How exactly adjustment of strophe duration (in the sense of the mechanism maintaining honesty of the signal) is related to signalling aggressiveness demands further testing as several alternatives are possible. For example, longer songs may signal higher male quality (Lambrechts and Dhondt 1987, Bischoff et al. 2009) or signal higher arousal by overlapping the song of a rival more easily (Dabelsteen et al. 1997). Future studies should aim to differentiate between possible mechanisms of how longer and shorter song versions may act and address all three criteria for aggressive signals presented by Searcy and Beecher (2009). Truly aggressive signals should occur frequently in aggressive context, predict an attack from the signaller and evoke the response of receivers.

It is worth to mention that responses in repeated experiments with the same individual may be affected by the order of treatments. Especially as we played back songs of the same neighbour twice and kept unchanged song traits that code individual identity (Osiejuk 2014). Here, we found in both experiments that males responded vocally stronger when first tested with longer songs. This result also supports the argument that longer songs are perceived as more aggressive, although we found no significant effect of treatment.

To conclude, we found that ortolan bunting males respond to simulated neighbour intrusion and that they respond differently to songs of different durations by approaching the loudspeaker playing longer songs more quickly. Similar observations have been made for other songbird species, and the opposite pattern of a stronger response to shorter songs was never reported (Nelson and Poesel 2012). The aforementioned review compiles research for 19 species of songbird with 12 of them eliciting a stronger approaching behaviour when longer song versions were presented. As we focused solely on song duration changes in our experimental manipulations, this study provides solid proof that differences in song strophe duration in a discontinuous singer might be informative and can be used as a specific response to potential rivals in acoustic territorial defence.
